# Partial‐EMT cell state correlates with single cell pattern of invasion in head and neck SCC keratinocytes

**DOI:** 10.1002/path.6454

**Published:** 2025-07-30

**Authors:** Pyung Hun Park, Lauren E Israel, Michael H Alexander, Grace Tartaglia, Harvey G South, Suhao Han, Joseph M Curry, Adam J Luginbuhl, Andrew P South

**Affiliations:** ^1^ Department of Dermatology and Cutaneous Biology, and Pharmacology, Physiology and Cancer Biology, Sidney Kimmel Medical College Thomas Jefferson University Philadelphia PA USA; ^2^ Department of Dermatology University of Wisconsin–Madison Madison WI USA; ^3^ Sydney Kimmel Cancer Center Thomas Jefferson University Philadelphia PA USA; ^4^ Department of Otolaryngology – Head and Neck Surgery Thomas Jefferson University Philadelphia PA USA; ^5^ University of Wisconsin Carbone Cancer Center Madison WI USA

**Keywords:** head and neck squamous cell carcinoma, invasion, invasive carcinoma, worst pattern of invasion, partial‐EMT, podoplanin, HIC1

## Abstract

The presence of a single metastatic lesion significantly decreases overall survival in patients with head and neck squamous cell carcinoma (HNSCC), and invasion of malignant keratinocytes is one of the initial steps required for HNSCC metastasis. Histological grading of tumor cell invasion predicts outcome in HNSCC, yet the molecular factors that determine the extent of invasion, and subsequent grading are not fully understood. Using a 3D organ culture model and multiple patient‐derived HNSCC keratinocytes representing all major anatomical subsites of the disease, we identified a range of cell states that represent a continuum of epithelial‐to‐mesenchymal (EMT) characteristics. We also demonstrated how these cell states change in response to TGF‐beta stimulation and co‐culture with cancer‐associated fibroblasts in organ cultures. Using 3D culture models that recapitulate the pattern of invasion seen in primary tumors from which the keratinocytes were derived, we identified distinct clusters of partial‐EMT marker expression in individual patient HNSCC keratinocyte populations. Partial‐EMT transcription factors were correlated with separate invasive characteristics, and we demonstrated that ZEB2 (a known EMT driver) and HIC1 (a novel EMT driver) are central nodes in HNSCC keratinocyte invasion. Collectively, our findings refine the concepts of partial‐EMT and tumor cell invasion, and identify potential therapeutic targets for future development. © 2025 The Author(s). *The Journal of Pathology* published by John Wiley & Sons Ltd on behalf of The Pathological Society of Great Britain and Ireland.

## Introduction

Head and neck cancer is the seventh most common malignancy worldwide, and head and neck squamous cell carcinoma (HNSCC) accounts for over 90% of these tumors [1]. While multimodal therapy including surgery, chemotherapy, radiotherapy, and immunotherapy exists to treat the disease, most patients who present with advanced tumors involving the lymph nodes or distant metastasis relapse within 3–5 years and experience a 5‐year survival rate of below 50% [2]. Of the factors that drive the initial steps of tumor dissemination, invasion of transformed keratinocytes into the underlying stromal tissue is arguably the most critical. The pattern of cell invasion, whether collectively, as large ‘buds’ or tumor islands, or as single cells has been used to predict disease progression in HNSCC for many years [3, 4, 5, 6, 7], and a greater understanding of this process will facilitate the development of better therapies for advanced HNSCC.

A hybrid tumor cell state, featuring both epithelial and mesenchymal characteristics (often referred to as partial epithelial‐to‐mesenchymal transition or p‐EMT), is associated with migration, invasion, metastasis, and even therapy resistance across multiple tumor types [8, 9, 10]. Early single‐cell analysis in HNSCC refined TCGA bulk RNA tumor classifications and identified p‐EMT as a cell state associated with lymph node‐positive tumors [9]. Cells with the highest p‐EMT state were identified at the leading edge of invading tumor islands, and refinements to bulk RNA tumor classifications identified metastasis‐associated ‘mesenchymal’ HNSCC as metastasis‐associated ‘basal’ HNSCC, with a high proportion of stromal content, indicating extensive tissue invasion [9]. These and a slew of published data strongly implicate the tumor microenvironment interaction as a major driver of tumor cell invasion, and multiple studies have demonstrated that cancer‐associated fibroblasts (CAFs), a major cellular component of the tumor microenvironment, promote tumor cell invasion in a variety of settings [11, 12, 13]. While CAFs and p‐EMT promote tumor cell invasion, the relationship between p‐EMT and tumor–CAF crosstalk in HNSCC invasion is less understood.

Activation of TGF‐beta (TGF‐β) signaling in tumor keratinocytes and the tumor microenvironment is associated with aggressive squamous cell carcinomas (SCCs), including immunosuppression and fibrosis [8, 14, 15, 16]. One paradox associated with the link between p‐EMT, metastasis, and TGF‐β signaling is the observation that components of the TGF‐β signaling pathway are frequently mutated in SCC, including HNSCC, and some tumor keratinocytes are unresponsive to TGF‐β ligand [17, 18]. Understanding the relationship between TGF‐β response, p‐EMT, and tumor cell invasion will contribute to the interpretation of somatic mutation data at diagnosis, an approach that is becoming more widespread with increased clinical uptake [19].

The aim of this study was to interrogate the relationship between HNSCC invasion patterns, TGF‐β, and p‐EMT using a diverse panel of primary tumor‐derived HNSCC keratinocyte populations in culture. We show that although the addition of CAFs to 3D organ culture models of HNSCC increases invasion regardless of tumor keratinocyte p‐EMT levels or response to TGF‐β ligand, p‐EMT correlates with a singular pattern of invasion in the presence of CAFs. Therefore, our study refines the concepts of p‐EMT in HNSCC tumor keratinocyte invasion.

## Materials and methods

A detailed description of the methods for 2D cell culture, imaging, cell proliferation assays, length‐to‐width calculations, western blotting, TGF‐β1 ELISA, quantitative PCR (qPCR), immunofluorescence, RNA and DNA sequencing, and transcription factor screening is provided in Supplementary materials and methods.

### Ethics approval and patient consent

Discarded tissue from patients undergoing SCC surgery was collected after informed consent under the Institutional Review Board (IRB)‐approved study ‘Squamous Cell Carcinoma Tumor Bank’ Control #15D.548.

### Cell culture

All primary keratinocytes (HN8, HN9, HN20, HN65, HN285, and HN379) and fibroblasts (HN9F) were derived from biopsies obtained during routine surgical resections from patients with HNSCC. Primary keratinocytes were established on a layer of mitotically inactivated NIH 3T3 cells and cultured in keratinocyte growth medium (KGM), consisting of Dulbecco's Modified Eagle Medium (DMEM) (Corning, Corning, NY, USA)/Ham's F‐12 media (Corning) (3:1 ratio) supplemented with 10% fetal bovine serum (Biowest USA Inc., Bradenton, FL, USA), 1% Pen‐Strep 100× solution (Corning), and growth factors at 37 °C and 5% CO_2_ as previously described [20]. All primary keratinocyte HNSCC populations became feeder‐independent after four passages. Primary fibroblasts were established as previously described [21] from the same tumor as the HN9 SCC keratinocytes were derived from and cultured in DMEM with 10% fetal bovine serum and 1% Pen‐Strep 100× solution, under the same incubation conditions. Cultures were routinely tested for mycoplasma using PCR, and only negative cells were used in this study.

### p‐EMT score of nine genes

Delta *CT* (Δ*CT*) values for each gene were calculated (*CT*_p‐EMT Gene_ − *CT*_GAPDH_ = Δ*CT*) before log transformation and normalization [2^(−Δ*CT*) * 100] to give the expression (*EXP*) value. The *EXP* value for each gene across all keratinocyte populations was used to define *EXP*_min_ and *EXP*_max_. The varying ranges of Δ*CT* and *EXP* for each of the nine p‐EMT genes were individually normalized {*N*_val_ = [(100 × *EXP*_min_) − *EXP*_max_]/99}, and the resulting *N*_val_ was used to determine an individual sample expression for each gene (for example, *EXP*_HN9(PDPN‐Ctrl)_ − *N*_val_ = p‐EMT_HN9(PDPN‐Ctrl)_). These individual normalized p‐EMT nine‐gene values were then averaged, with all values converted to the range of 0.1–10, to represent the p‐EMT score used in this study.

### Organ culture

To prepare organotypic cultures, 3.5 volumes of collagen type I, 5 mg/ml (R&D Systems, Minneapolis, MN, USA), were combined with 1 volume of 10× DMEM (Corning) and 1 volume of FBS, and approximately 0.1 volume of 1 m NaOH was added to bring the mixture to a neutral pH. To this mixture, 3.5 volumes of Cultrex (a Matrigel® equivalent, 10 mg/ml, R&D Systems) and 1 volume of fibroblasts (1.5 × 10^6^ cells/ml) or 1 volume of KGM medium alone were added. All preparations were performed on ice using chilled pipettes and containers. If used, human TGF‐β1 recombinant protein at 5 ng/ml (Cell Signaling Technology, Inc., Danvers, MA, USA) or SB431542 at 10 μm (Selleckchem, Houston, TX, USA) was added to the gel mixture prior to polymerization. 1.5 ml of the gel mixture was added to one well of a 12‐well plate and allowed to polymerize for 30 min at 37 °C. KGM media (see above) supplemented with l‐ascorbic acid (5 μmol) (Wako Chemicals, Richmond, VA, USA) and aprotinin (20 mg/ml) (Sigma‐Aldrich, St Louis, MO, USA) were used for all subsequent steps. After polymerization, 1.5 × 10^5^ fibroblasts were seeded in 2 ml of media and allowed to settle for 3 h before 2.0 × 10^6^ keratinocytes were added. After 48 h, the organ culture was transferred to a six‐well plate, where it was placed on top of a nylon mesh (Millipore Sigma, Burlington, MA, USA) and a stainless steel mesh to create an air‐to‐liquid interface, where the culture is not completely immersed in media, exposing the keratinocyte layer to air (see supplementary material, Movie S1). Organ cultures were maintained for 14 days, with media changes every other day before harvesting.

### Invasion index, worst pattern of invasion (WPOI), and single pattern of invasion (SPOI)

Cytokeratin and DAPI immunofluorescence images of 5 μm FFPE‐embedded organ culture sections (see Supplementary materials and methods) were obtained using a DeltaVision Ultra (GE Healthcare, Chicago, IL, USA) using 20× magnification. Triplicate images were acquired from duplicate or triplicate independent 3D culture experiments from the center of the gel, one‐quarter from the left, and one‐quarter from the right of each gel, generating nine images for each independent experiment. These images were then quantified and averaged to provide a single sample size (*n*). The threshold function in ImageJ (NIH, Bethesda, MD, USA) was used to highlight tumor keratinocytes, and an invasion index was calculated as previously described [22] by multiplying the area, number of tumor islands, and depth of tumor invasion.

The worst pattern of invasion (WPOI) was based on previously reported methods [4] and graded as follows: WPOI type 1: tumor invasion with a broad pushing front; WPOI type 2: ‘finger‐like’ pushing pattern of tumor invasion; WPOI type 3: invasive islands of tumors with more than 15 cells per island; WPOI type 4: invasive islands of tumors with fewer than 15 cells per island, which may also include long strands of infiltrating tumor cells; and WPOI type 5: 1 mm or greater of normal tissue between tumor islands.

The single pattern of invasion (SPOI) was calculated in the same manner as the invasion index, with the following modifications: instead of using area and depth, SPOI was calculated by dividing the number of singular tumor islands (islands with less than 15 nuclei) by the number of clustered tumor islands (islands with 15 or more nuclei). If no singular tumor islands were present, the value was recorded as 0.

### Statistical analyses

Significance was determined using GraphPad Prism (RRID:SCR_002798). A *p* value < 0.05 was considered statistically significant: *p < 0.05, **p < 0.01, ***p < 0.001, and ****p < 0.0001. All data are specified as normalized or not in the figure legends. Description of statistical tests, including the number of independent experiments (*n*) performed either in duplicate or in triplicate, units, central mean, and SEM dispersion, is given in the figures or figure legends. Statistical tests were performed as parametric or non‐parametric when appropriate. Multiple paired Student's *t*‐tests were conducted on qPCR values, p‐EMT scores, length‐to‐width ratio (LWR), and western blotting bar graph plots. Correlation analysis was performed to calculate the *r* value and *p* value for each correlation plot. Simple linear regression was applied to each correlation plot for a best‐fit line. The ‘Identify Outliers’ function of Prism (under ‘Analyze’) was used to remove outliers for SPOI fold‐change comparisons.

## Results

### Primary HNSCC keratinocyte morphology in 2D culture correlates with p‐EMT and invasion into 3D organ cultures

**Figure 1:**
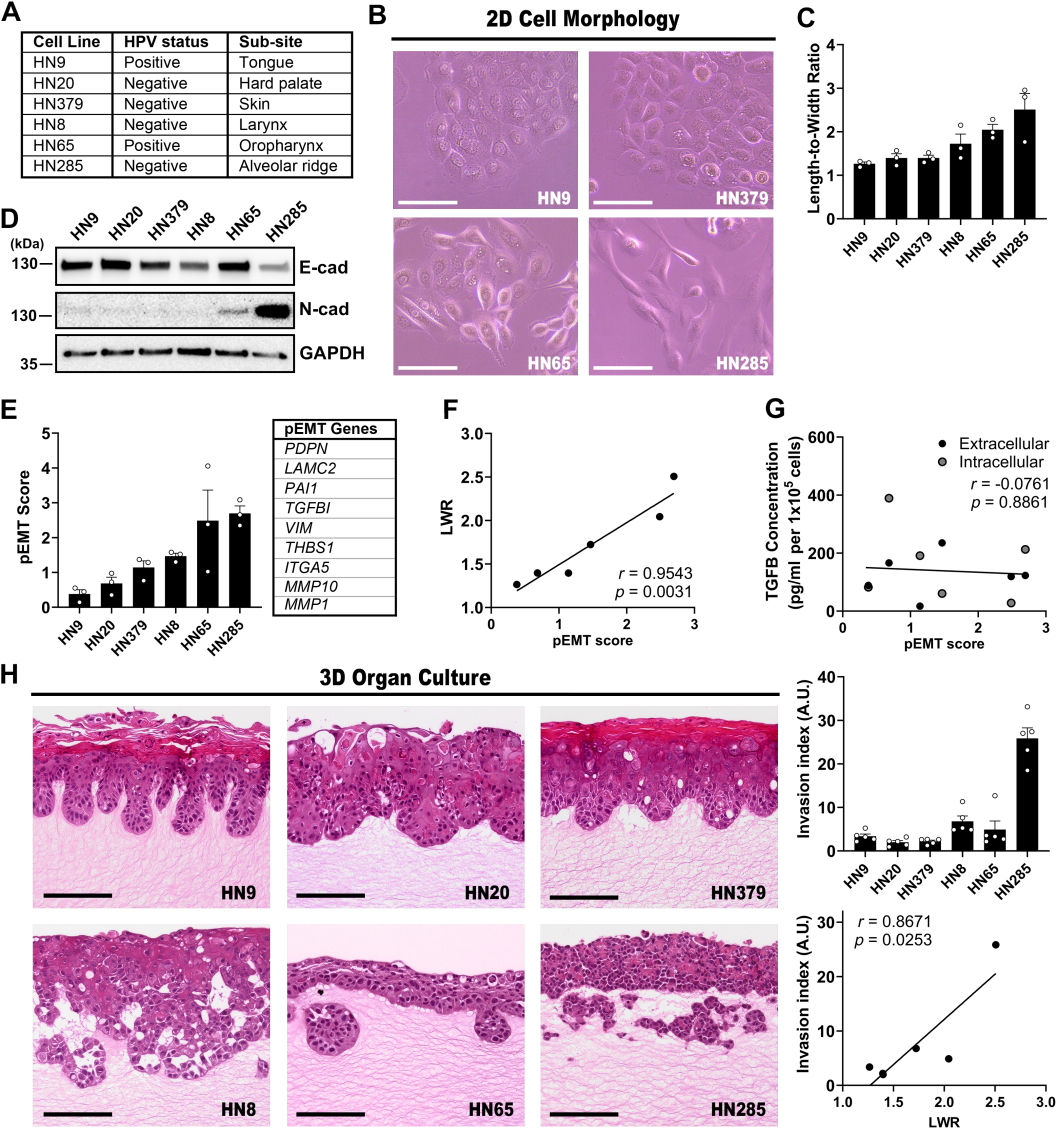
Primary HNSCC keratinocyte morphology in 2D culture correlates with p‐EMT score and invasion into 3D organotypic matrices. (A) HPV status and sub‐site of origin for HNSCC keratinocytes. (B) 2D cell culture morphology representing different states of tumor keratinocytes. HN9 and HN379 keratinocytes show a compact epithelial pattern, while HN65 and HN285 keratinocytes show an elongated, mesenchymal pattern. Images were captured with EVOS at 20× magnification. Scale bars, 100 μm. (C) Length‐to‐width ratio (LWR) as calculated from 2D culture images. The cells with a value closer to 1 represent a more cuboidal epithelial cell compared with a more mesenchymal‐like phenotype when the value is higher (*n* = 3; 50 cells measured for each cell line). (D) Western blotting analysis of baseline E‐cadherin and N‐cadherin in HNSCC keratinocytes. (E) Baseline p‐EMT scores derived from normalized qPCR values of nine p‐EMT genes. Each dot represents the average normalized value of the nine p‐EMT genes (*n* = 3). (F) Correlation between LWR and p‐EMT scores from panels C and E. (G) Correlation between extracellular and intracellular TGF‐β (TGFB) and p‐EMT scores (ns). (H) The left panels show representative images of hematoxylin and eosin (H&E)‐stained 3D organ cultures. Scale bar, 150 μm. The bar chart on the right shows invasion index calculated from 3D organ culture images (*n* = 5). The bottom‐right graph shows the correlation between the invasion index and LWR.

We established a panel of patient SCC tumor keratinocyte populations (HN8, HN9, HN20, HN65, HN285, and HN379) from resections of HPV‐positive and HPV‐negative tumors located at various anatomical sites (Figure 1A and supplementary material, Table S1). HNSCC keratinocytes displayed a range of morphological features when grown at sub‐confluent densities: from classical keratinocyte colonies with defined cell–cell connections through to individual phase‐bright cells with mesenchymal appearance (Figure 1B). The LWR of HNSCC keratinocytes grown at sub‐confluent densities reflected these morphological differences (Figure 1B,C). All HNSCC keratinocytes expressed the epithelial marker E‐cadherin (Figure 1D) and generated tight cell–cell connections when grown at confluency, displaying a classical keratinocyte sheet phenotype (supplementary material, Figure S1A). The two HNSCC keratinocyte populations with the greatest LWR also expressed consistently detectable levels of the mesenchymal cadherin N‐cadherin (Figure 1D).

To determine whether morphology in 2D culture correlated with p‐EMT status, we calculated an *in vitro* p‐EMT score based on mRNA levels (as determined by qPCR) of nine genes previously identified as classical markers of p‐EMT from multiple published studies [23, 24, 25, 26, 27, 28, 29, 30, 31, 32]. A strong correlation (*r* = 0.95, *p* = 0.003) between LWR and p‐EMT score was observed (Figure 1E,F) which was independent of endogenous levels of TGF‐β1, a known inducer of p‐EMT in 2D culture conditions (Figure 1G and supplementary material, Figure S1B,C).

We next examined HNSCC keratinocyte invasion into 3D organ cultures composed of collagen I and Matrigel™ and observed a range of invasion (quantified from measurements of tumor cell area, depth, and islands of invasion as previously described [22]). LWR correlated with invasion into 3D organ cultures (Figure 1H), and p‐EMT score showed a positive, yet non‐significant correlation with invasion (*p* = 0.1; supplementary material, Figure 1D).

### TGF‐β1 exposure increases p‐EMT score and invasion into 3D organ cultures in TGF‐β‐responsive HNSCC keratinocytes

**Figure 2:**
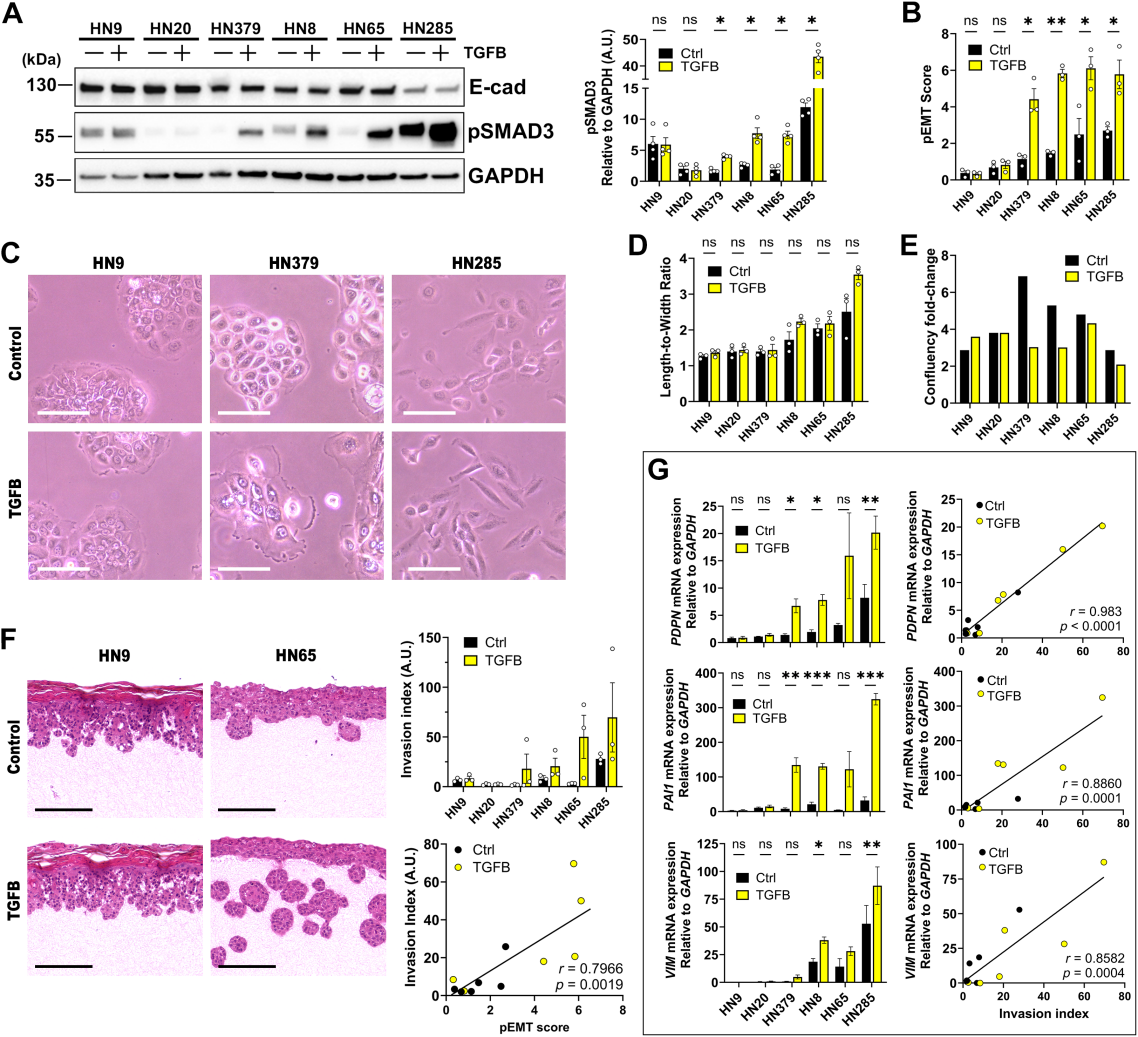
TGF‐β ligand increases p‐EMT score and invasion into 3D organotypic cultures in TGF‐β‐responsive HNSCC keratinocytes. (A) Western blotting showing phosphorylated SMAD3 (pSMAD3) and E‐cadherin (E‐cad) levels following treatment with (+) or without (−) TGF‐β (5 ng/ml) for 48 h. Bar chart shows quantification of pSMAD3 levels relative to GAPDH (*n* = 4). (B) Bar chart showing p‐EMT score of control and TGF‐β‐treated keratinocytes as determined by qPCR (*n* = 3). (C) Representative images of control and TGF‐β‐treated keratinocytes. Cells were seeded and treated with TGF‐β (5 ng/ml) or vehicle control for 48 h. Scale bars, 100 μm. (D) Length‐to‐width ratio (LWR) of keratinocytes 48 h after TGF‐β (5 ng/ml) or control (Ctrl) treatment (*n* = 3). (E) Cell confluency using IncuCyte live‐cell analysis was used to determine the proliferation of HNSCC cells treated with TGF‐β (5 ng/ml) or control for 48 h, comparing IncuCyte images from 48 h and 0 h. (F) Representative images of TGF‐β‐non‐responsive (HN9) and TGF‐β‐responsive (HN65) keratinocytes in 3D organ culture. Scale bars: 200 μm. Bar chart shows invasion index of 3D organ culture of control and TGF‐β‐treated samples, and correlation plot shows correlation between the invasion index and p‐EMT score of control and TGF‐β‐treated samples. (G) Relative mRNA expression of *PDPN*, *VIM*, and *PAI1* normalized to *GAPDH* (bar charts, left), and their correlation with invasion index for each HNSCC cell line (plots, right). *p < 0.05, **p < 0.01, ***p < 0.001.

We next investigated the relationship between TGF‐β response, p‐EMT, and invasion, since TGF‐β is a known driver of p‐EMT and is associated with increased migration and invasion [9, 33]. Exposing cultured HNSCC keratinocytes to 5 ng/ml TGF‐β1 for 48 h and measuring the levels of phosphorylated SMAD3 identified two populations that were not responsive to TGF‐β (HN9 and HN20) and a range of stimulation (2‐ to 4‐fold) in the remaining four populations (Figure 2A). In keeping with literature identifying TGF‐β as a major driver of p‐EMT, addition of TGF‐β1 increased p‐EMT scores for the four TGF‐β‐responsive HNSCC keratinocyte populations but did not affect the two non‐responsive populations (Figure 2B). Interestingly, LWR varied in TGF‐β‐responsive HNSCC keratinocytes after ligand exposure, with a reduction in one population (HN379; Figure 2C,D). In addition, a reduction in proliferation after TGF‐β1 exposure was observed in only two populations (HN379 and HN8) while unaffecting others (Figure 2E). Regardless of this variation in LWR and proliferation, TGF‐β stimulation increased invasion in 3D organ culture for all four TGF‐β‐responsive HNSCC keratinocyte populations and correlated with p‐EMT score when all six HNSCC keratinocyte populations were compared (Figure 2F), suggesting that TGF‐β‐responsive keratinocytes invade collagen/Matrigel™ organ cultures and this invasion can be enhanced further by the addition of TGF‐β1. In those HNSCC populations unresponsive to TGF‐β, there is limited invasion into organ cultures, which remains unchanged after the addition of TGF‐β1. Comparing all populations, p‐EMT score is increased in TGF‐β‐responsive HNSCC keratinocytes and can be further increased with the addition of TGF‐β1.

### *PDPN* , *PIA1*, and *VIM* mRNA correlate with invasion into 3D organotypic cultures

Next, we revisited each of the nine genes comprising our p‐EMT score to determine whether 3D organ culture invasion is influenced by individual gene expression. Endogenous *PDPN*, *PAI1*, and *VIM* mRNA expression in 2D culture showed a significant (*p* < 0.001), positive (*r* > 0.85) correlation with invasion into 3D organ cultures (Figure 2G and supplementary material, Figure S2).

### Fibroblast tumor cell interactions drive 3D organotypic HNSCC invasion regardless of p‐EMT levels or TGF‐β response

**Figure 3:**
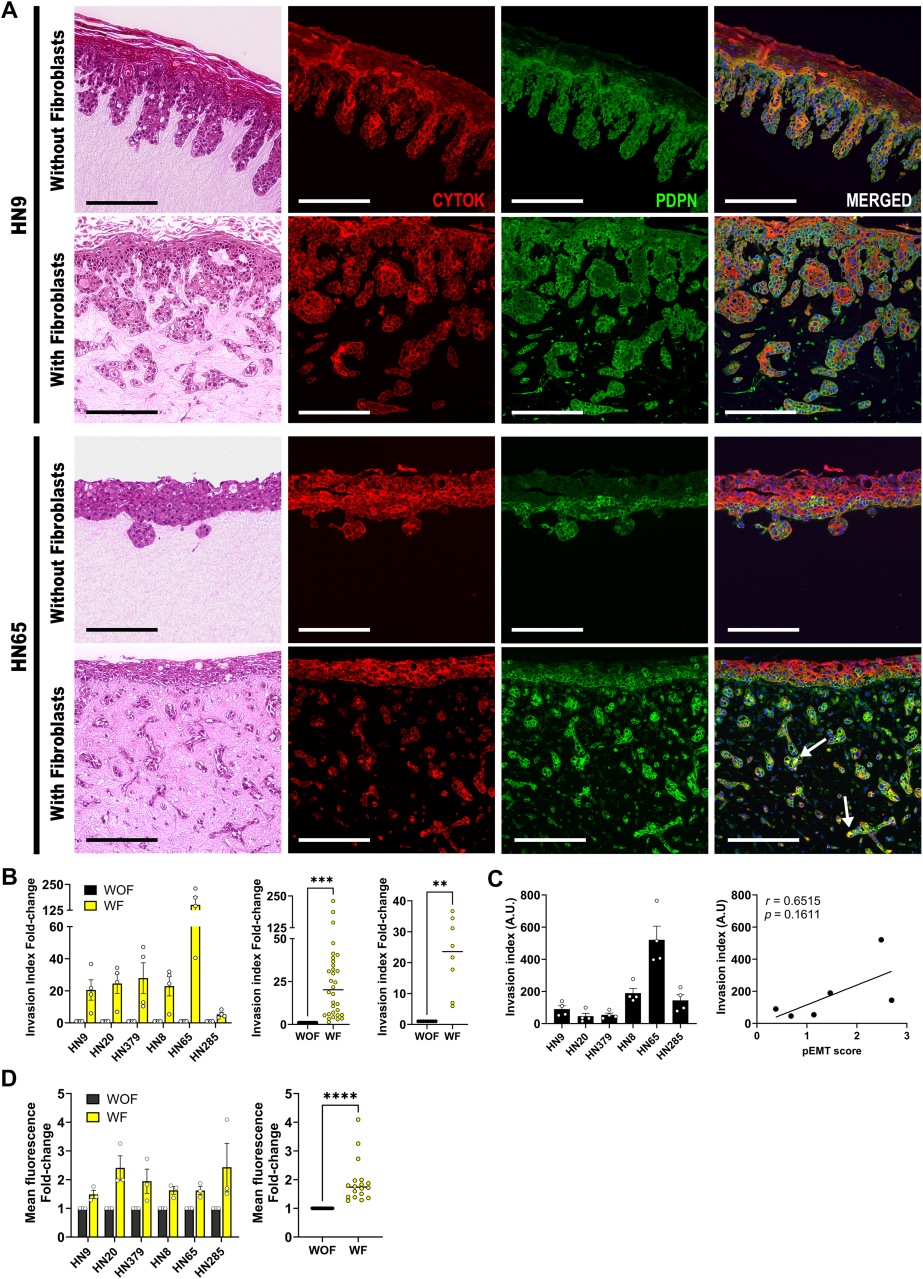
Fibroblast tumor cell interactions drive 3D organotypic HNSCC invasion regardless of TGF‐β response. (A) Representative H&E and immunofluorescence staining images (red = cytokeratin; green = podoplanin; blue = nucleus, DAPI) of HN9 (TGF‐β non‐responsive) and HN65 (TGF‐β responsive) 3D organ cultures. Scale bars, 200 μm. (B) Bar chart showing invasion index fold‐change of 3D organ culture, with (WF) and without (WOF) fibroblasts (left panel) (*n* = 4 for each keratinocyte). The middle panel shows plot of aggregate invasion index across all HNSCC cell lines, comparing organ cultures with fibroblasts (WF) or without fibroblasts (WOF) (*n* = 24). The right panel shows a similar plot with only TGF‐β‐non‐responsive HNSCC cell lines (*n* = 8). (C) Bar chart shows invasion index in the presence of fibroblasts (*n* = 4, left panel), and correlation plot shows the relationship between invasion index and p‐EMT score (right panel). (D) Bar chart shows quantification of PDPN expression in organ cultures with fibroblasts (WF) or without fibroblasts (WOF) after immunostaining (left panel) (*n* = 3 for each keratinocyte). The plot on the right shows the same data in aggregate comparing the result with and without fibroblasts (*n* = 18). **p < 0.01, ***p < 0.001, ****p < 0.0001.

Since tumor cell invasion is greatly influenced by the presence of fibroblasts and other cells within the tumor microenvironment [34], we introduced HNSCC fibroblasts into the collagen I/Matrigel™ organ cultures and measured invasion after 14 days. As anticipated from previous work utilizing different cell lines [11], the presence of fibroblasts in 3D organ cultures significantly increased the extent of invasion, in most cases by an order of magnitude, regardless of whether the HNSCC keratinocytes were TGF‐β‐responsive (Figure 3A,B). TGF‐β ligand had no effect on invasion in the presence of fibroblasts (supplementary material, Figure S3A,B), in contrast to TGF‐β‐responsive HNSCC keratinocytes in 3D organ culture without fibroblasts (Figure 2E,F). On the other hand, inhibition of TGF‐β using the TGF‐β receptor type 1 inhibitor SB431542 reduced the invasion of TGF‐β‐responsive HNSCC keratinocytes into 3D organ cultures, with little change to proliferation in 2D culture (supplementary material, Figure S3A–C).

Whilst there was no significant correlation between p‐EMT score in 2D culture and absolute values for invasion in 3D organ cultures supplemented with fibroblasts, a positive relationship was observed with invasion in the three HNSCC keratinocyte populations with the highest p‐EMT score (Figure 3C).

### Fibroblast tumor cell interactions increase PDPN expression

Immunofluorescence examination of the nine p‐EMT markers chosen to represent our initial p‐EMT score revealed a unilateral increase in PDPN expression with the presence of fibroblasts in 3D organ cultures [Figure 3A (right panels) and Figure 3D]. Addition of TGF‐β1 or inhibition of TGF‐β in 3D organ cultures with fibroblasts had little effect on PDPN expression, with the exception of HN379, which showed a measurable increase in PDPN with TGF‐β1 addition and a reduction of PDPN expression in HN8, HN65, and HN285 with TGF‐β inhibition (supplementary material, Figure S3D).

### Pattern of 3D organ culture invasion in the presence of fibroblasts reflects the pattern of invasion in primary tumors

**Figure 4:**
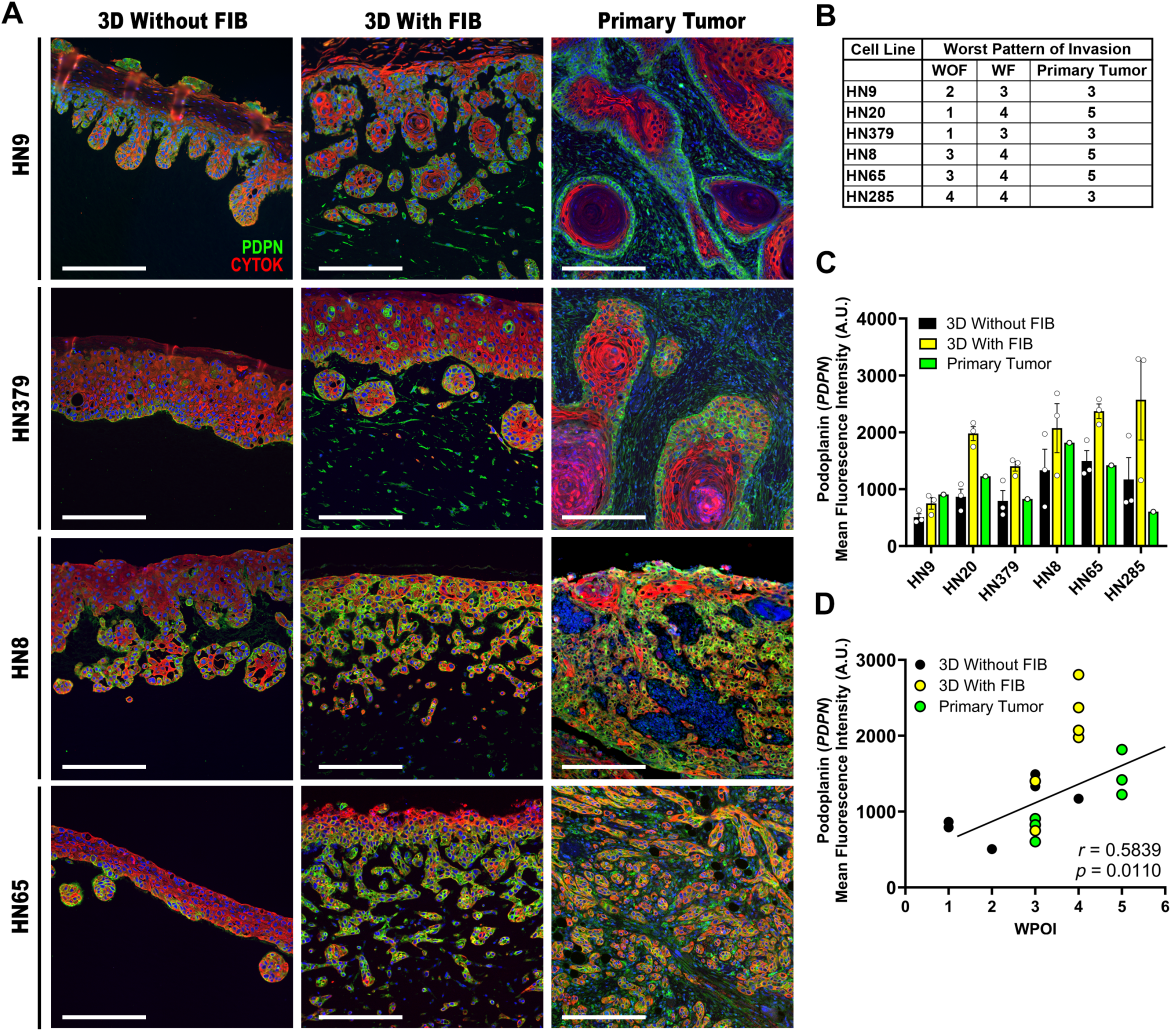
Fibroblast tumor cell interactions drive the pattern of HNSCC invasion reflecting patient primary tumor morphology. (A) Immunofluorescence images of HNSCC keratinocyte cell lines in 3D organ culture, without or with fibroblasts (FIB), and corresponding primary tumor from which the keratinocyte cell line was derived. Podoplanin (PDPN) is visualized in green, cytokeratin (CYTOK) in red, and the nucleus in blue (DAPI). Scale bars, 200 μm. (B) Worst pattern of invasion (WPOI) values for 3D organ cultures and primary tumor of origin for the HNSCC lines HN8, HN9, HN20, HN65, HN285, and HN379. (C) Quantification of PDPN expression from immunofluorescence images of 3D organ cultures and primary tumors. (D) Correlation between WPOI and PDPN expression in 3D cultures (without fibroblasts and with fibroblasts) and primary tumor.

Using the WPOI score, which classifies tumor histology based on the presence of invasive islands, their size, and distance from the tumor mass [4, 26], we compared 3D organ cultures, with and without fibroblasts, with the primary tumors of origin for all six HNSCC keratinocyte populations. Except for HN285 (where WPOI did not change when comparing 3D organ cultures with or without fibroblasts, and where available histology blocks contained only a small portion of the tumor to analyze), the addition of fibroblasts to 3D organ cultures increased the WPOI score in line with the primary tumor when compared with 3D organ cultures without fibroblasts (Figure 4A,B). Expression levels of PDPN in primary tumors were generally lower than those in organ cultures but still correlated with WPOI (Figure 4C,D).

### Pattern of 3D organ culture invasion correlates with endogenous p‐EMT score in the presence of fibroblasts

**Figure 5:**
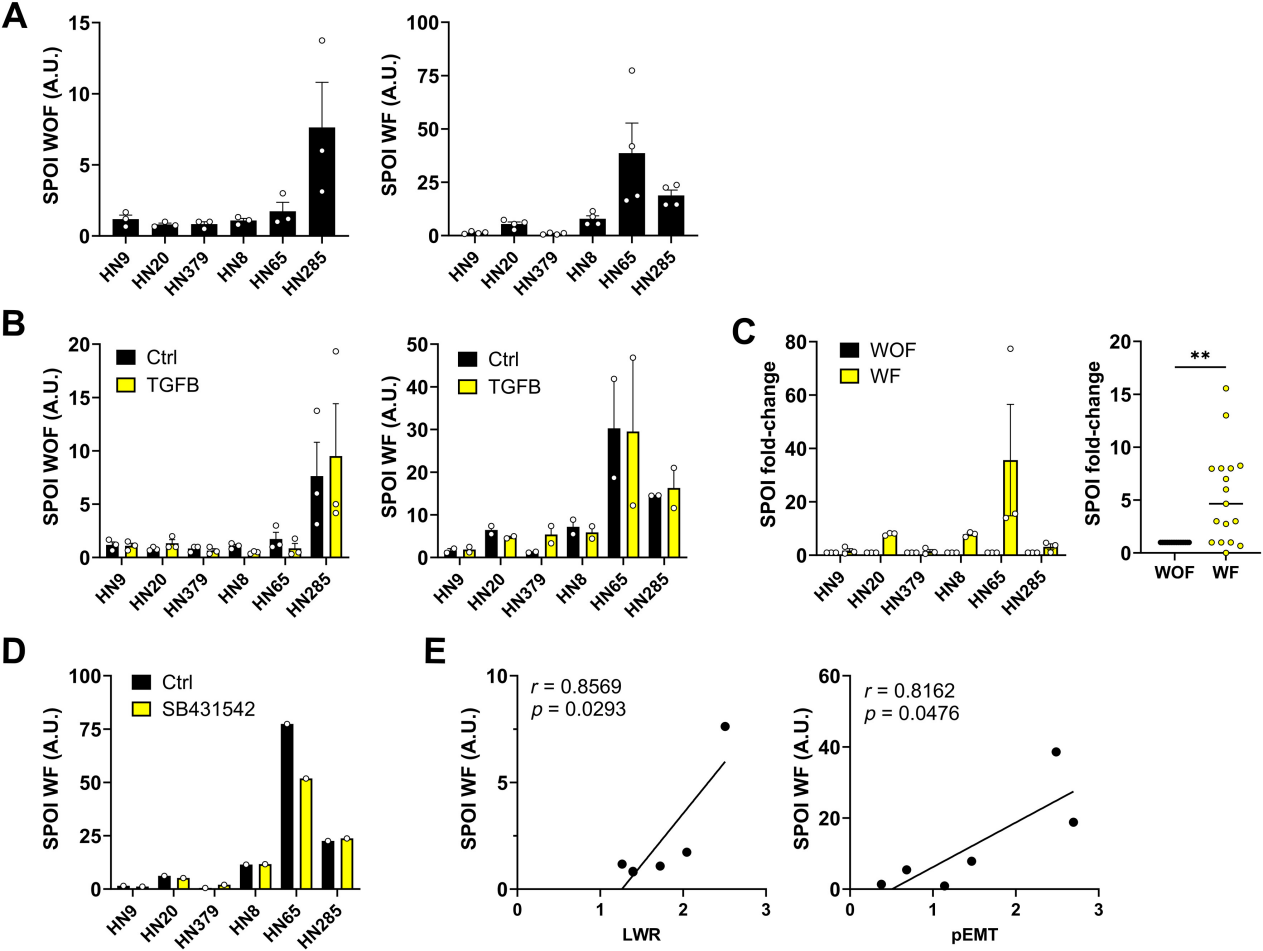
Singular pattern of invasion is increased by fibroblast tumor cell interaction and correlates with endogenous tumor cell p‐EMT. (A) Single pattern of invasion (SPOI) of individual HNSCC cell lines in 3D organ culture without fibroblasts (WOF) (left, *n* = 3) and with fibroblasts (WF) (right, *n* = 4). (B) SPOI in 3D organ cultures WOF (left, *n* = 3) or WF (right, *n* = 2) treated with either TGF‐β or vehicle controls. (C) Bar chart shows SPOI fold‐change between WOF and WF (*n* = 3), and the plot on the right shows aggregate data excluding a single outlier (*n* = 17). (D) Bar chart shows SPOI in 3D organ cultures treated with SB431542 or vehicle control (Ctrl) (*n* = 1). (E) Correlation plots showing the relationship between SPOI WOF versus LWR (left) and SPOI WF versus p‐EMT (right). **p < 0.01.

WPOI identifies five categories: 1, being absence of invasion; 2, exhibiting a ‘finger‐like’ pushing pattern of invasion; 3, showing large invasive islands (>15 cells); 4, measuring single cellular islands of invasion (less than 15 cells); and 5, measuring any invasion greater than 1 mm in distance from the primary tumor mass [4, 26]. Since this distance, 1 mm, is at the limit of the depth of 3D organ cultures, the highest WPOI score *in vitro* can only be 4. With this in mind, we measured islands of single cellular invasion (<15 cells) as a factor of total invasive islands in our 3D organ cultures to derive a singular pattern of invasion, termed SPOI. SPOI was unaffected by the addition or inhibition of TGF‐β and correlated with the invasion index in 3D organ cultures with or without fibroblasts. LWR correlated with SPOI in 3D organ cultures without fibroblasts, and p‐EMT correlated with SPOI in 3D organ cultures with fibroblasts (Figure 5). These data suggest that while the addition of fibroblasts to 3D organ cultures greatly increases overall invasion, increased endogenous p‐EMT increases the singular pattern of invasion independent of TGF‐β stimulation (Figure 5B).

### Transcription factors associated with invasion segregate dependent on tumor keratinocyte–fibroblast interactions

To further refine factors that contribute to HNSCC keratinocyte invasion into 3D organ cultures, we performed whole‐exome DNA sequencing and RNA sequencing of sub‐confluent replicate cultures of HNSCC keratinocytes used in this study.

Whole‐exome sequencing identified a homozygous 21‐bp in‐frame deletion of exon 6 of the *TGFBR2* gene in HN9, which likely explains the lack of response to TGF‐β ligand. For HN20, the other TGF‐β‐unresponsive cell line, heterozygous single‐nucleotide mutations of unknown significance were identified in the untranslated regions of *SMAD4* and *TGFB2*. None of the other lines harbored mutations in TGF‐β receptor, ligand, or *SMAD* genes, but they did exhibit the expected array of known HNSCC driver gene mutations (supplementary material, Figure S4A). Copy number analysis identified structural change in all six HNSCC cell lines used in this study (supplementary material, Figure S4B).

**Figure 6:**
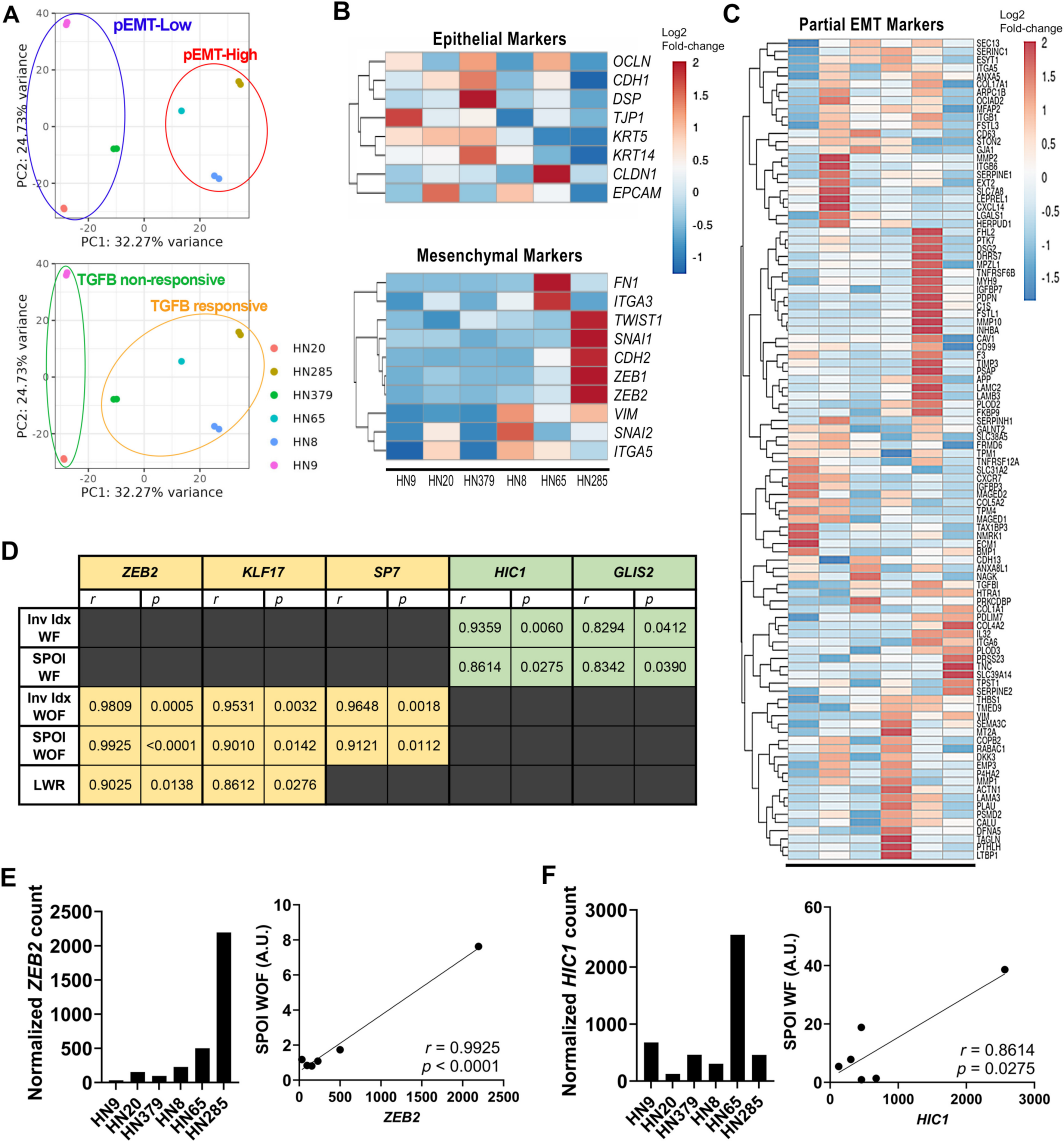
Transcriptomic analysis of HNSCC keratinocytes identifies varying cell states of p‐EMT and individual transcription factors that correlate with invasion. (A) Principal component analysis of RNA‐sequencing data from sub‐confluent cultures of HNSCC cell lines. The top panel highlights samples by p‐EMT scores (p‐EMT‐high versus p‐EMT‐low) and the bottom panel highlights TGF‐β‐responsive and TGF‐β‐non‐responsive samples. (B) Heatmap of epithelial and mesenchymal marker expression across the six HNSCC cell lines used in this study. (C) Heatmap of the top 100 significant p‐EMT genes previously identified by Puram *et al* [9] clustered by mRNA expression levels across the six HNSCC cell lines used in this study. (D) Table showing *r* and *p* values comparing transcription factors identified in common between VIM, PAI, and PDPN with invasion index and SPOI values from 3D organ culture experiments with (WF) or without (WOF) the addition of fibroblasts. (E) Relationship between *ZEB2* mRNA expression levels (left bar chart) and SPOI without fibroblasts (WOF; right plot). (F) Relationship between *HIC1* mRNA expression levels (left bar chart) and SPOI with fibroblasts (WF; right plot).

For RNA sequencing, principal component analysis showed no close grouping of patient keratinocyte populations as expected from a diverse group of tumors, with respect to HPV status and anatomical location, HNSCC keratinocyte populations loosely clustered based on TGF‐β response, and levels of p‐EMT markers, primarily driven by principle component 1 (Figure 6A). Quantification of classical epithelial and mesenchymal marker levels from the RNA‐sequencing data showed a similar level of heterogeneity (Figure 6B). While previously classified markers of p‐EMT suggested some stratification of HNSCC keratinocyte populations, no clear correlations with any of our previous measurements, including TGF‐β response, LWR, or measures of invasion, were observed (Figure 6C).

Since mRNA expression of *PDPN*, *VIM*, and *PAI1* correlated with invasion into 3D organ cultures (Figure 2G), we examined the relationship between 40 transcription factors, which associated with each of these three gene promoters. From this analysis, two groups of transcription factors emerged that correlated with organ culture invasion and SPOI. Levels of *KLF17*, *SP7*, and *ZEB2* correlated with invasion and SPOI in 3D organ cultures in the absence of fibroblasts, whereas levels of *HIC1* and *GLIS2* correlated with SPOI in 3D organ cultures in the presence of fibroblasts (Figure 6D–F), suggesting that these transcription factors may influence either endogenous invasive properties or those that respond to external stimuli.

## Discussion

As the hybrid EMT, or p‐EMT, cell state in HNSCC is associated with metastatic disease and therapy resistance, our study sought to understand the relationship between p‐EMT and invasion in primary tumor keratinocytes isolated from a diverse group of HNSCC tumors. Our initial focus on a nine‐gene p‐EMT signature identified tumor populations that associated with the continuum of enrichment in epithelial markers through to mesenchymal markers (Figure 1E), and these data were in part confirmed by RNA sequencing, highlighting classical markers of epithelial and mesenchymal cell types (Figure 6B). Examination of a broader p‐EMT gene list, previously identified from single‐cell analysis of tumor keratinocytes *in vivo* [9], revealed that distinct clusters of p‐EMT markers dominate individual HNSCC keratinocyte populations. This suggests that p‐EMT gene expression levels in culture segregate into modules specific to individual tumors. We further examined this clustering by segregation of ten distinct modules (supplementary material, Figure S5A) and pathway analysis. Individual modules were generally too small to identify pathways of significance. However, grouping modules based on heatmap clustering and p‐EMT status showed the following hits: TP53 signaling was enriched in low p‐EMT modules; ECM interaction, HPV infection, and PI3K–Akt signaling were enriched in mixed and high p‐EMT modules; and focal adhesion and TGF‐β signaling were enriched in high p‐EMT modules (supplementary material, Figure S5B). Further work will be needed to dissect how the tumor microenvironment influences changes in these modules and whether these changes further influence tumor invasion.

One marker widely used to identify p‐EMT in multiple tumor types is PDPN [23, 35] and our data demonstrated that PDPN expression increased with invasion in all HNSCC keratinocyte populations tested here (Figures 2D, 3D, and 4D), and this increase was independent of overall p‐EMT score or TGF‐β response, although PDPN expression was increased by the addition of TGF‐β in TGF‐β‐responsive keratinocytes (Figure 2G). These observations suggest that PDPN, a component of focal adhesions necessary for cellular motility [36], may be associated with tumor cell invasion, independent of overall p‐EMT levels. Whether PDPN facilitates, or is a consequence of, invasion remains to be determined.

With regard to TGF‐β, our data agreed with previous observations that TGF‐β promotes collective cell migration and invasion in both cancer and development [37, 38], but also identified tumor–CAF crosstalk as the dominant driver of invasion regardless of endogenous p‐EMT or ability to respond to TGF‐β.

Previous studies of p‐EMT in primary murine and human tissues demonstrated intratumor heterogeneity, in terms of the extent of cellular p‐EMT state within sub‐populations of tumor cells, revealing substantial variation in the expression of p‐EMT markers [39, 40]. In this study, we analyzed each separate patient HNSCC population as a whole, predominantly in 2D culture, and therefore likely missed any level of intratumor heterogeneity if present. However, our measurements are primarily of cells in 2D monoculture (under arguably homogeneous conditions) and heterogeneity can be observed in 3D organ cultures, from immunofluorescence analysis of individual proteins (Figure 3A), and we interpret our data in 2D culture as the baseline for a given population which will be modified by 3D context and cell–cell interaction. The clear similarities between 3D organ cultures with fibroblasts and primary tumor tissues (Figure 4) supports this belief and the distinct segmentation of populations with regard to the large list of p‐EMT markers identified by Puram *et al* [9] identifies each HNSCC population being dominated by a distinct subset of these markers comparing across all six populations, suggesting that baseline levels or the state of p‐EMT is dictated by an individual patient's tumor (Figure 6C). We predict that this baseline, or 2D culture cell state, would be changed upon interaction with the tumor microenvironment and further work with these and other cell lines will determine if this holds true.

The WPOI [26] is a histological grading system that measures the depth and pattern of invasion and has been used to strongly predict disease progression in HNSCC for many years [4]. This system measures the type of invasion as well as the presence of tumor ‘buds’ (or islands), scored as either ‘singular’ or ‘larger’ islands of tumor invasion either smaller than or equal to/greater than 15 cells, respectively. The scale ranges from 1 to 5, with a score of 3 indicating larger invasive islands, a score of 4 indicating singular (<15 cells) invasive islands, and a score of 5 indicating any invasive island greater than 1 mm away from the primary tumor mass. Although our 3D organ cultures were not able to consistently accommodate tumor islands greater than 1 mm from the tumor mass, and we were unable to score above 4, we demonstrated that the addition of fibroblasts increased WPOI closer to that observed in the primary tissue from which the cells were derived (Figure 4B). As a result, we devised a scoring system based purely on the proportion of single islands of invasion (<15 cells), termed singular pattern of invasion (SPOI). We observed that SPOI was elevated by the presence of fibroblasts and that baseline p‐EMT correlated with the level of SPOI, suggesting that while the presence of fibroblasts dictated the extent of invasion, the pattern of singular invasive islands was dependent on the extent of baseline p‐EMT, irrespective of TGF‐β stimulation (Figure 5). Whether this baseline represents positional information retained from the primary tumor, driven by epigenetic changes, or whether the baseline represents combinatorial effects of somatic mutation, or a combination of both, remains to be elucidated.

Our EMT transcription factor analysis, focusing on those transcription factors reported to interact with the promoters of *PDPN*, *VIM*, and *PAI1*, identified two groups that correlated with the level of invasion of HNSCC keratinocytes with or without fibroblasts (Figure 6), suggesting that specific transcription factors influence endogenous invasion while others are more important for tumor cell–CAF interactions. This agrees with data in breast cancer identifying ZEB2 as being important for tumor cell migration and invasion and ZEB1 being more important for tumor cell growth/colony formation [39].

While the ZEB family of transcription factors has a clear role in p‐EMT, invasion, and colony formation, the role of HIC1 (hypermethylated in cancer‐1), a member of the POZ family of zinc‐finger transcription factors and originally identified as a putative tumor suppressor [41], is less clear. Loss of *HIC1* has been associated with prostate cancer and induction of EMT [42], while *HIC1* recently identified a group of mesenchymal progenitors in the developing limb [43], suggesting that HIC1 may drive a mesenchymal phenotype rather than inhibit one in certain cell types. In addition to this, *HIC1* has also been implemented in immune cell differentiation [44], and it seems that cellular context is a critical determinant of HIC1 function, likely driven by its observed regulation through epigenetic processes such as methylation [41]. In addition to this, *HIC1* is a p53 target gene [41] and we note that greater expression of *HIC1* was detected in our *TP53* wild‐type HNSCC lines (Figure 6F and supplementary material, Figure S4A). A single study has implicated p53 mutation status in the expression of a truncated form of HIC1 [45], not investigated by our study, and it is clear that further work is necessary to dissect the regulation and role of HIC1 in HNSCC.

In conclusion, our study refines concepts of p‐EMT and tumor cell invasion in HNSCC and identifies *PDPN* and *HIC1* as potential therapeutic targets for future development.

## Author contributions statement

APS and PHP were responsible for manuscript writing and editing. APS, PHP, MHA, GT and SH were responsible for data collection. APS, PHP, LEI and HGS analyzed the data. AJL and JMC were responsible for patient sample preparation. APS was responsible for the study concept and critical review. All authors reviewed and approved the final manuscript.

## Supporting information


Supplementary materials and methods
**Figure S1.** HNSCC keratinocyte morphology at confluence and endogenous TGF‐β1 levels
**Figure S2.** Relative mRNA expression of *LAMC2*, *TGFBI*, *TBHS1*, *MMP1*, *MMP10*, and *ITGA5* normalized to *GAPDH* across all HNSCC keratinocyte lines (bar charts) and correlation plots comparing invasion index with relative mRNA expression
**Figure S3.** HNSCC 3D organ culture invasion and PDPN expression after TGF‐β stimulation or inhibition
**Figure S4.** HNSCC driver gene mutations and DNA copy number variations
**Figure S5.** p‐EMT gene expression across HNSCC keratinocytes identifies modules of transcription

**Table S1.** Patient details (provided in a separate Excel file)

**Movie S1.** HN9 3D organ culture day 14 (provided in a separate file)

## Data Availability

The data that support the findings of this study are available from the corresponding author upon reasonable request.
