# Supplementary material for: Partial‐EMT cell state correlates with single cell pattern of invasion in head and neck SCC keratinocytes
Source: J Pathol. 2025 Jul 30;267(1):92–104. doi: 10.1002/path.6454 (PMC12313228; doi:10.1002/path.6454)
Supplement: Supplementary file 1 — Supplementary materials and methods Figure S1. HNSCC keratinocyte morphology at confluence and endogenous TGF‐β1 levels Figure S2. Relative mRNA expression of LAMC2, TGFBI, TBHS1, MMP1, MMP10, and ITGA5 normalized to GAPDH across all HNSCC keratinocyte lines (bar charts) and correlation plots comparing invasion index with relative mRNA expression Figure S3. HNSCC 3D organ culture invasion and PDPN expression after TGF‐β stimulation or inhibition Figure S4. HNSCC driver gene mutations and DNA copy number variations Figure S5. p‐EMT gene expression across HNSCC keratinocytes identifies modules of transcription [file PATH-267-92-s003.doc]

**Partial-EMT cell state correlates with single cell pattern of invasion in head and neck SCC keratinocytes**

PH Park *et al. J Pathol* <https://doi.org/10.1002/path.6454>

**Supplementary materials and methods**

**Supplementary Figures S1–S5**

**Supplementary Table S1** (provided in a separate Excel file)

**Supplementary Movie S1** (provided in a separate file)

Reference numbers refer to the main text list

**Supplementary materials and methods**

*2D cell culture imaging*

Morphological observation of primary keratinocytes was performed using an EVOS Cell Imaging System (Thermo Fisher Scientific, Waltham, MA, USA) with 10× magnification in a bright-field microscope. Images were cropped to equal sizes, ensuring accurate scale bar representation in the figures.

*Length-to-width ratio* (*LWR*) *calculations*

2D cell morphology images were acquired using the EVOS Cell Imaging System (Thermo Fisher Scientific) at 10× magnification in bright-field view. Each independent experiment (sample size, *n*) included triplicate images for analysis. For each image, 50 cells were measured for length (widest part of the cell body) and width (largest perpendicular measurement to length). The length-to-width ratio (LWR) was calculated by dividing length by width. For TGF-β-induced morphological observations, cells were treated with 5 ng/ml human TGF-β1 recombinant protein in keratinocyte growth medium (KGM) for 48 h. For SB431542-induced morphological observations, cells were treated with 10 µm SB431542 in KGM for 48 h.

*Cell proliferation assay*

Keratinocytes were seeded at 8.0 × 10⁴ cells per well in 12-well plates. After 24 h, cells were treated with 5 ng/ml human TGF-β1 recombinant protein (Cell Signaling Technology, Inc., Danvers, MA, USA) or 10 µm SB431542 (Selleckchem, Houston, TX, USA) in KGM for 48 h. Cells were monitored using an IncuCyte® S3 system (Essen BioScience, Ann Arbor, MI, USA), capturing images (10× objective) every 4 h for 48 h in four separate regions per well. Cell proliferation was calculated by dividing the final cell confluency by the initial cell confluency.

*Western blotting*

Keratinocytes were seeded at 3.0 × 10^5^ cells per well in six-well plates and, after 24 h, treated with human TGF-β1 recombinant protein at 5 ng/ml in KGM for 48 h. Cells were then lysed in RIPA buffer (Thermo Fisher Scientific) supplemented with protease and phosphatase inhibitors (Thermo Fisher Scientific) on ice and incubated for 10 min. After the incubation, cells were collected with a cell scraper and transferred to a 1.5 ml Eppendorf tube for centrifugation at 13,300 rpm and 4 °C. The supernatant was collected, and protein concentration was measured using the Pierce Rapid Fold BCA Protein Assay Kit (Thermo Fisher Scientific). Samples were loaded onto 8–16% acrylamide gels (Bio-Rad, Hercules, CA, USA). For protein loading, 15 μg of protein was loaded for E-cadherin, GAPDH, and phospho-SMAD3, and 25 μg of protein was loaded for N-cadherin. Primary antibodies included phospho-SMAD3 (Abcam, Cambridge, UK; ab52903) at 1:1,000 dilution; E-cadherin (BD Biosciences, Franklin Lakes, NJ, USA; 610181) at 1:2,000 dilution; N-cadherin (BD Biosciences; 610920) at 1:700 dilution; and GAPDH (Santa Cruz Biotechnology Inc., Dallas, TX, USA; sc-365062) at 1:2,500 dilution.

Proteins were transferred onto a nitrocellulose membrane with a Bio-Rad Trans-Blot Turbo (Bio-Rad), blocked in PBS-0.1% Tween (Thermo Fisher Scientific) with 5% milk or 5% BSA (Thermo Fisher Scientific) according to the primary antibody’s requirements, and incubated overnight at 4 °C with the primary antibody. After incubation with IgG-HRP conjugated secondary antibody (Cell Signaling Technology, Inc.,), the membrane was incubated with SuperSignal West Pico Plus Chemiluminescent Substrate (Thermo Fisher Scientific) and exposed to the FluorChem R exposure system (Bio-Techne, Minneapolis, MN, USA). Western blotting signals were analyzed using ImageJ, RRID:SCR_003070 (https://scicrunch.org/resolver/RRID:SCR_003070). Each protein was quantified relative to GAPDH as the loading control.

*TGF-β1 ELISA*

Keratinocytes were seeded at 3.0 × 10^5^ cells per well in six-well plates, and KGM was replaced 24 h later. Keratinocytes were cultured for a further 48 h. Lysates and media were collected according to the manufacturer’s guidelines (R&D Systems, DB100C). The protocol was followed before using the FlexStation 3 (Molecular Devices, San José, CA, USA) to measure absorbance. TGF-β1 concentration was further normalized by cell number.

*Quantitative PCR*

Keratinocytes were seeded at 3.0 × 10^5^ cells per well in six-well plates and, after 24 h, treated with human TGF-β1 recombinant protein at 5 ng/ml in KGM for 48 h. Total RNA was harvested using RNA STAT-60 (AMSBIO, Abingdon, UK), following the manufacturer’s protocol. RNA extractions were quantified using the Qubit RNA Broad Range Assay Kit (Thermo Fisher Scientific), and 1.5 μg of RNA was used for cDNA synthesis using the SuperScript III First-Strand Synthesis System (Invitrogen, Waltham, MA, USA; 12574026). Primers designed by Origene (Rockville, MD, USA) were used, including GAPDH (forward: GTCTCCTCTGACTTCAACAGCG; reverse: ACCACCCTGTTGCTGTAGCCAA), *PDPN* (forward: GTGCCGAAGATGATGTGGTGAC; reverse: GGACTGTGCTTTCTGAAGTTGGC), *LAMC2* (forward: TACAGAGCTGGAAGGCAGGATG; reverse: GTTCTCTTGGCTCCTCACCTTG), *PAI1* (forward: CTCATCAGCCACTGGAAAGGCA; reverse: GACTCGTGAAGTCAGCCTGAAAC), *VIM* (forward: AGGCAAAGCAGGAGTCCACTGA; reverse: ATCTGGCGTTCCAGGGACTCAT), *TGFBI* (forward: GGACATGCTCACTATCAACGGG; reverse: CTGTGGACACATCAGACTCTGC), *THBS1* (forward: GCTGGAAATGTGGTGCTTGTCC; reverse: CTCCATTGTGGTTGAAGCAGGC), *MMP10* (forward: TCCAGGCTGTATGAAGGAGAGG; reverse: GGTAGGCATGAGCCAAACTGTG), *MMP1* (forward: ATGAAGCAGCCCAGATGTGGAG; reverse: TGGTCCACATCTGCTCTTGGCA), and *ITGA5* (forward: GCCGATTCACATCGCTCTCAAC; reverse: GTCTTCTCCACAGTCCAGCAAG).
qPCR was performed with the desired target primers and cDNA using SYBR Select Master Mix (Thermo Fisher Scientific). A QIAgility robot (Qiagen, Redwood City, CA, USA) was used to pipette primers, samples, and Master Mix for amplification, and the Rotor-Gene Q (Qiagen) was used for amplification and measurement. Experiments were performed in duplicate due to the highly accurate robot pipetting. The qPCR cycles were as follows: initial holds at 50 °C for 2 min and 95 °C for 2 min, 35 cycles of annealing (95 °C for 15 s, 60°C for 60 s), followed by 95 °C for 15 s, 60 °C for 15 s, and 95 °C for 15 s.

*Immunofluorescence* (*IMF*)

Organ culture or patient tissue samples were fixed for 24 h in 10% buffered formalin, paraffin-embedded (FFPE), and cross-sectioned at 5 μm. Samples were baked overnight and rehydrated, followed by antigen retrieval in citrate buffer. Slides were permeabilized in 0.1% Triton X-100 in PBS for 10 min at room temperature, followed by blocking in 5% BSA in PBS with 0.1% Tween-20 for 1 h at room temperature. Slides were incubated with primary antibodies overnight at 4 °C, including cytokeratin AE1/AE3 (Millipore Sigma, Burlington, MA, USA; MAB4312) at 1:700, podoplanin (R&D Systems, Minneapolis, MN, USA; AF3670) at 10 µg/ml, and vimentin (Santa Cruz Biotechnology Inc., sc-6260) at 1:500. Slides were washed three times in PBS prior to secondary antibody application.

Secondary antibodies were applied after washing: anti-sheep Alexa Fluor 488 (Thermo Fisher Scientific, A-11015) at 1:400, anti-mouse Alexa Fluor 594 (Thermo Fisher Scientific, A-21203) at 1:500, and anti-rabbit Alexa Fluor 488 (Abcam, Cambridge, UK; ab150077) at 1:400 for 1 h at room temperature. Slides were washed three times in PBS prior to mounting with DAPI Fluoromount-G (SouthernBiotech, Birmingham, AL, USA). Slides were imaged using a DeltaVision Ultra (GE Healthcare, Chicago, IL, USA) and an A1R Microscope (Nikon, Tokyo, Japan). Three replicate images from each independent experiment (sample size, *n*) were taken for analysis. The histogram feature in ImageJ was used to measure the front edge of the epithelial layer and tumor islands, with background noise subtracted for normalization to quantify staining.

*DNA sequencing*

DNA was isolated from HN keratinocytes and corresponding fibroblasts (isolated from the same sample to serve as germline reference) using DNeasy Blood and Tissue Kits (Qiagen), and equal ng were used to generate libraries using Illumina DNA Prep with Enrichment and the Illumina Exome Panel v1.2 following the manufacturer’s protocol (Illumina, Inc., San Diego, CA, USA). Equal nm of each library were pooled together and sequenced on a NovaSeq 6000 (Illumina) with 150 × 2 pair-end cycles. FASTQ files were processed using the Illumina BaseSpace DRAGEN Somatic v4.3.13 Application as tumor–normal pairs, using the Grch38 version of the human genome in the Map/Align + Somatic Small Variant Caller Pipeline Configuration. Variants were called within DRAGEN Somatic and annotated with both the built-in DRAGEN Somatic Nirvana Variant Annotator and the GATK Funcotator using Funcotator datasources v1.7.20200521s. Annotations were analyzed after VCF Filtering in Python. Copy number analysis was performed using the GATK Best Practices Workflow: Somatic copy number variant discovery under the latest GATK docker image (v4.6.1.0).

*RNA sequencing*

Sequencing libraries were generated with equal ng of each sample (prepared as described for qPCR) using the Illumina Stranded Total RNA Prep with Ribo-Zero Plus (Illumina) following the manufacturer’s protocol. In brief, Ribo-Zero Plus rRNA removal mix depleted abundant rRNA and globin mRNA in human samples and the remaining mRNA and long non-coding RNAs were targeted for library preparation. Equal nm of each library were pooled together and sequenced on a NovaSeq 6000 (Illumina), with 100 × 2 pair-end cycles along with 1% Phix. Raw FASTQ files were preprocessed to remove adapter sequences using Cutadapt version 4.6 [44,45], with the appropriate adapter sequences for forward and reverse reads. Post-trimming, the quality of the processed reads was assessed using FastQC (RRID:SCR_014583) version 0.12.1 (<http://www.bioinformatics.babraham.ac.uk/projects/fastqc>/), and the results were aggregated using MultiQC (RRID:SCR_014982) version 1.19 [46,47]. Trimmed and quality-controlled reads were aligned to the reference genome using STAR version 2.7.11b [48]. Transcript abundance quantification was performed using Salmon version 1.10.1 [49]. Differential gene expression analysis was conducted using the DESeq2 package [50] in R. Principal component analysis (PCA) was performed using the pcaExplorer package [51], and volcano plots were generated with the EnhancedVolcano package [52], focusing on protein-coding genes with specific significance thresholds. Functional enrichment analysis was conducted using ShinyGO version 0.77 [last accessed 19 August 2024] [53], considering genes with adjusted *p* values less than 0.05, absolute log_2_ fold-change greater than or equal to 1, and base mean expression levels exceeding 100. Data have been deposited in NCBI’s Gene Expression Omnibus and are accessible through GEO Series accession number GSE277508 (https://www.ncbi.nlm.nih.gov/geo/query/acc.cgi?acc=GSE277508).

*Transcription factor screening*

GeneHancer [54] was used to identify promoter/enhancer elements associated with *PDPN*, *VIM*, and *PAI1*. *PDPN* had 51 transcription factor binding sites derived from GeneHancer (GH Identifier GH01J013583). *VIM* had two promoter/enhancer elements, GH10J017226 and GH10J017212, with 271 and 137 transcription factor binding sites, respectively. *PAI1* had two promoter/enhancer elements, GH07J101116 and GH07J101153, with 259 and 144 transcription factor binding sites, respectively. We identified 40 overlapping transcription factor binding sites from GH01J013583, GH10J017226, GH10J017212, GH07J101116, and GH07J101153.

*Pathway analysis of p-EMT modules*

KEGG pathway analysis was performed using the ShinyGO version 0.82 tool ([https://bioinformatics.sdstate.edu/go/](https://nam10.safelinks.protection.outlook.com/?url=https%3A%2F%2Furldefense.com%2Fv3%2F__https%3A%2Fbioinformatics.sdstate.edu%2Fgo%2F__%3B!!Mak6IKo!NfzRJ0yOuVwzVIbxSghII0D9L9SX_XKQj270FmPrEvjTuEj1PoBoYHCMc2nQMlYnqGVIlRlh8m3IrU7KnrRVXUi5U4Bv_mY-pd7lmg$&data=05|02|PyungHun.Park@jefferson.edu|953c28eeeb494762bfe208dd88e954fc|55a89906c710436bbc444c590cb67c4a|0|0|638817257943685147|Unknown|TWFpbGZsb3d8eyJFbXB0eU1hcGkiOnRydWUsIlYiOiIwLjAuMDAwMCIsIlAiOiJXaW4zMiIsIkFOIjoiTWFpbCIsIldUIjoyfQ%3D%3D|0|||&sdata=5Kmt3Zk4jAYht15rwBhANS%2BQCfnL5%2B3ikJA4pZthOko%3D&reserved=0); last accessed 23 April 2025) using gene sets grouped by low p-EMT, mixed p-EMT, and high p-EMT modules. Low p-EMT module genes included *CD63*, *STON2*, *GJA1*, *MMP2*, *ITGB6*, *SERPINE1*, *EXT2*, *SLC7A8*, *P3H2*, *CXCL14*, *GALS1*, *HERPUD1*, *SLC31A2*, *ACKR3*, *IGFBP3*, *MAGED2*, *COL5A2*, *TPM4*, *MAGED1*, *TAX1BP3*, *NMRK1*, *ECM1*, and *BMP1*. Mixed p-EMT module genes included *SEC13*, *SERINC1*, *ESYT1*, *ITGA5*, *ANXA5*, *COL17A1*, *ARPC1B*, *OCIAD2*, *MFAP2*, *ITGB1*, *FSTL3*, *SERPINH1*, *GALNT2*, *SLC38A5*, *FRMD6*, *TPM1*, *TNFRSF12A*, *CDH13*, *ANXA8L1*, *NAGK*, *TGFBI*, *HTRA1*, *CAVIN3*, *COL1A1*, *COPB2*, *RABAC1*, *DKK3*, *EMP3*, *P4HA2*, and *MMP1*. High p-EMT module genes included *FHL2*, *PTK7*, *DSG2*, *DHRS7*, *MPZL1*, *TNFRSF6B*, *MYH9*, *IGFBP7*, *PDPN*, *C1S*, *FSTL1*, *MMP10*, *INHBA*, *CAV1*, *CD99*, *F3*, *TIMP3*, *PSAP*, *APP*, *LAMC2*, *LAMB3*, *PLOD2*, *FKBP9*, *PDLIM7*, *COL4A2*, *IL32*, *ITGA6*, *PLOD3*, *PRSS23*, *TNC*, *SLC39A14*, *TPST1*, *SERPINE2*, *THBS1*, *TMED9*, *VIM*, *SEMA3C*, *MT2A*, *ACTN1*, *LAMA3*, *PLAU*, *PSMD2*, *CALU*, *GSDME*, *TAGLN*, *PTHLH*, and *LTBP1*].


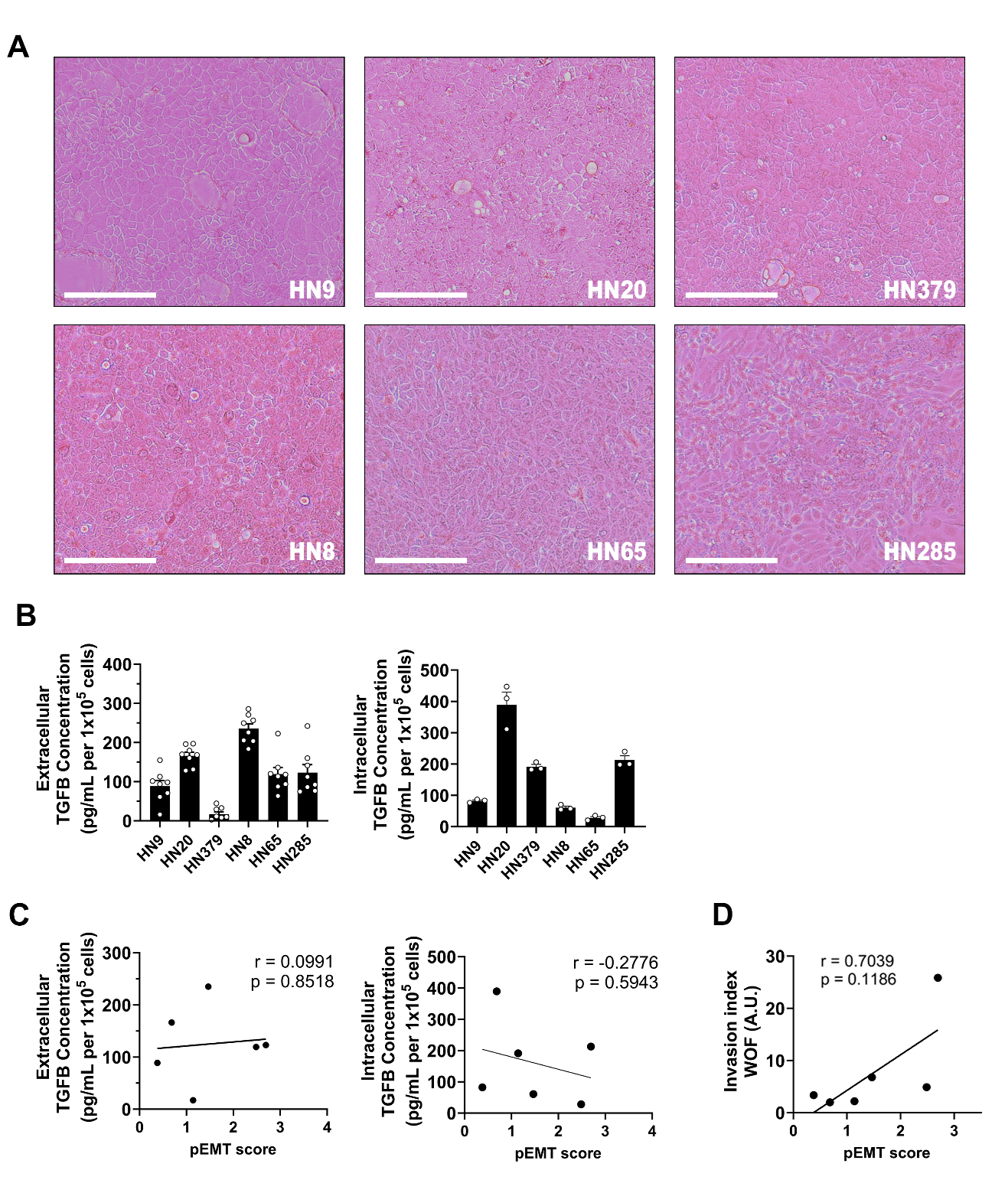


**Figure S1.** **HNSCC keratinocyte morphology at confluence and endogenous TGF-β1 levels.** (A) 2D cell culture morphology of confluent HN keratinocyte lines. Keratinocytes were seeded (1 × 10^6^ cells per well) in six-well plates and allowed to grow for 72 h. Scale bar: 200 µm. (B) Extracellular and intracellular TGF-β1 (TGFB) concentration as determined by ELISA. Keratinocytes were seeded (3 × 10^5^ cells per well) in a six-well plate 24 h prior to changing KGM media. Cells were cultured for a further 48 h, and media and cells were collected and used in ELISA assays. TGF-β1 concentration was normalized to background media and cell number (extracellular *n*= 8, intracellular *n*= 3). (C) Correlation plot of extracellular TGF-β1 and p-EMT score (left panel, ns). Correlation plot of intracellular TGF-β1 and p-EMT score (right panel, ns). (D) Correlation plot of invasion index without fibroblast (WOF) and p-EMT score.


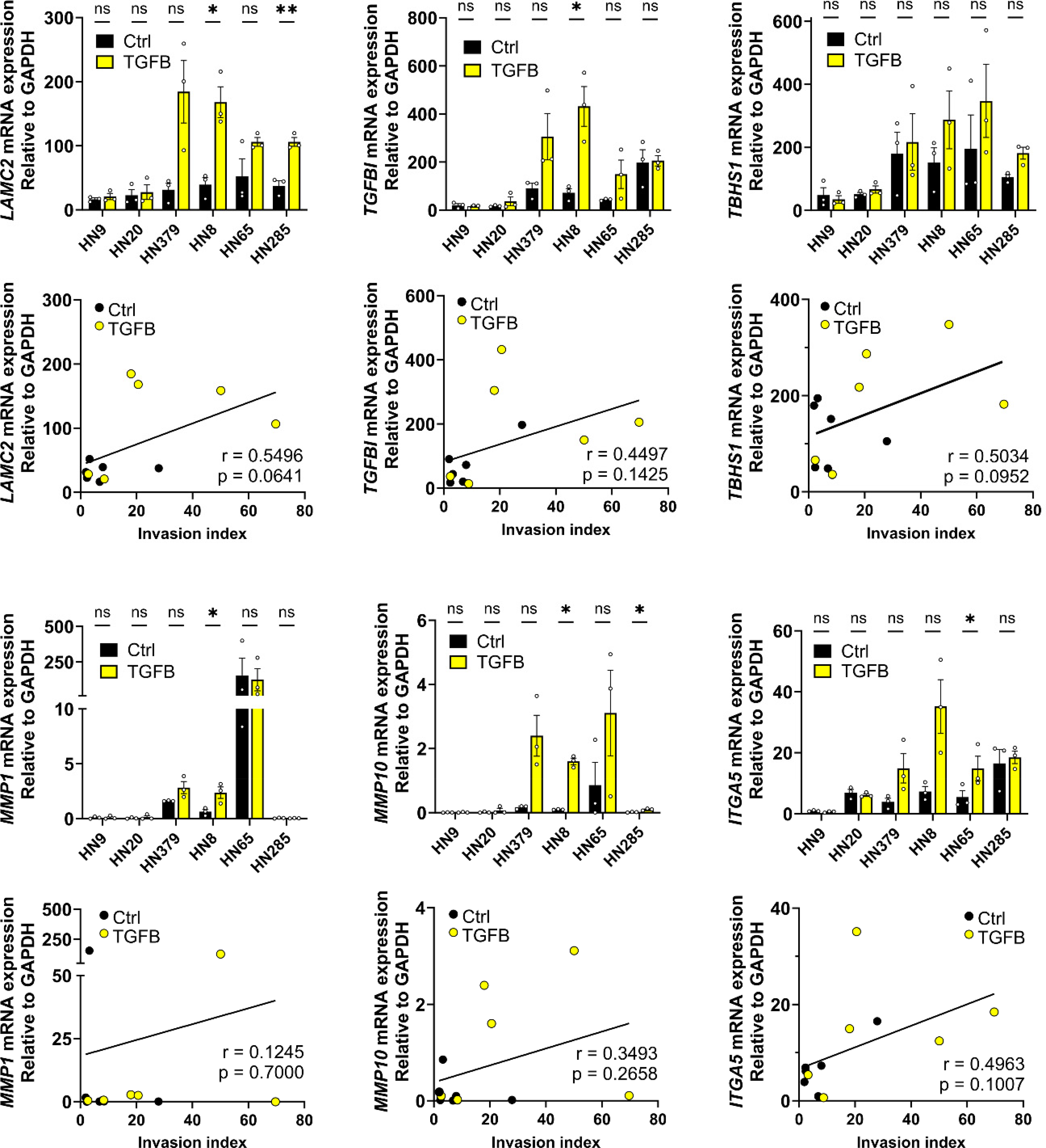


**Figure S2.** **Relative mRNA expression of *LAMC2*, *TGFBI*, *TBHS1*, *MMP1*, *MMP10*, and *ITGA5* normalized to *GAPDH* across all HNSCC keratinocyte lines used in this study with and without TGF-β1 (TGFB) addition (bar charts) and correlation plots** **comparing invasion index with relative mRNA expression.** **p* < 0.05, ***p* < 0.01.


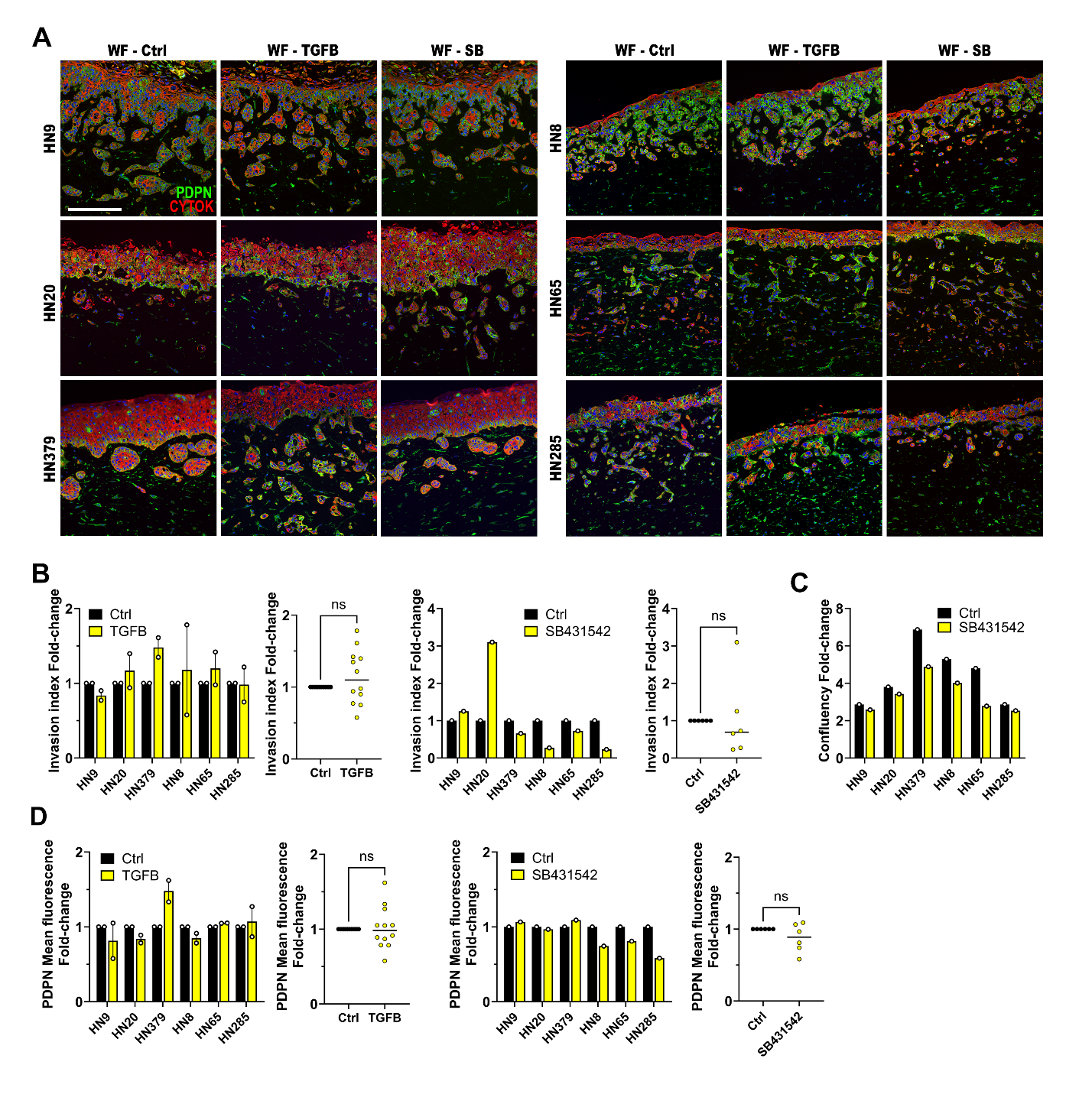


**Figure S3.** **HNSCC 3D organ culture invasion and PDPN expression after TGF-β stimulation or inhibition.** (A) Immunofluorescence signals (PDPN = green, CYTOK = red, DAPI = blue) captured with widefield fluorescence microscopy from 3D organ cultures of all keratinocytes (HN9, HN20, HN379, HN8, HN65, and HN285) with fibroblasts (WF) over 14 days with either TGF-β1 (TGFB) (5 ng/ml), SB431542 (SB) (10 µm), or vehicle control. Scale bar: 200 µm. (B) Quantification of invasion of HN keratinocytes into 3D organ cultures with fibroblasts shown in panel A. Far left panel shows individual keratinocyte invasion index fold-change comparing Ctrl and TGF-β1-treated samples (left, *n*= 2) or comparing Ctrl and SB-treated samples (right, *n*= 1). (C) Cell proliferation was measured from confluency determined using the IncuCyte live-cell analysis instrument. Cells were treated with SB (10 µm) for 48 h. (D) Quantification of PDPN signal in 3D organ cultures with fibroblasts shown in panel A. Far left panel shows individual keratinocyte PDPN mean fluorescence fold-change between Ctrl and TGF-β1-treated samples (*n*= 2) (left panels) and between Ctrl and SB-treated samples (*n*= 1) (right panels).

**
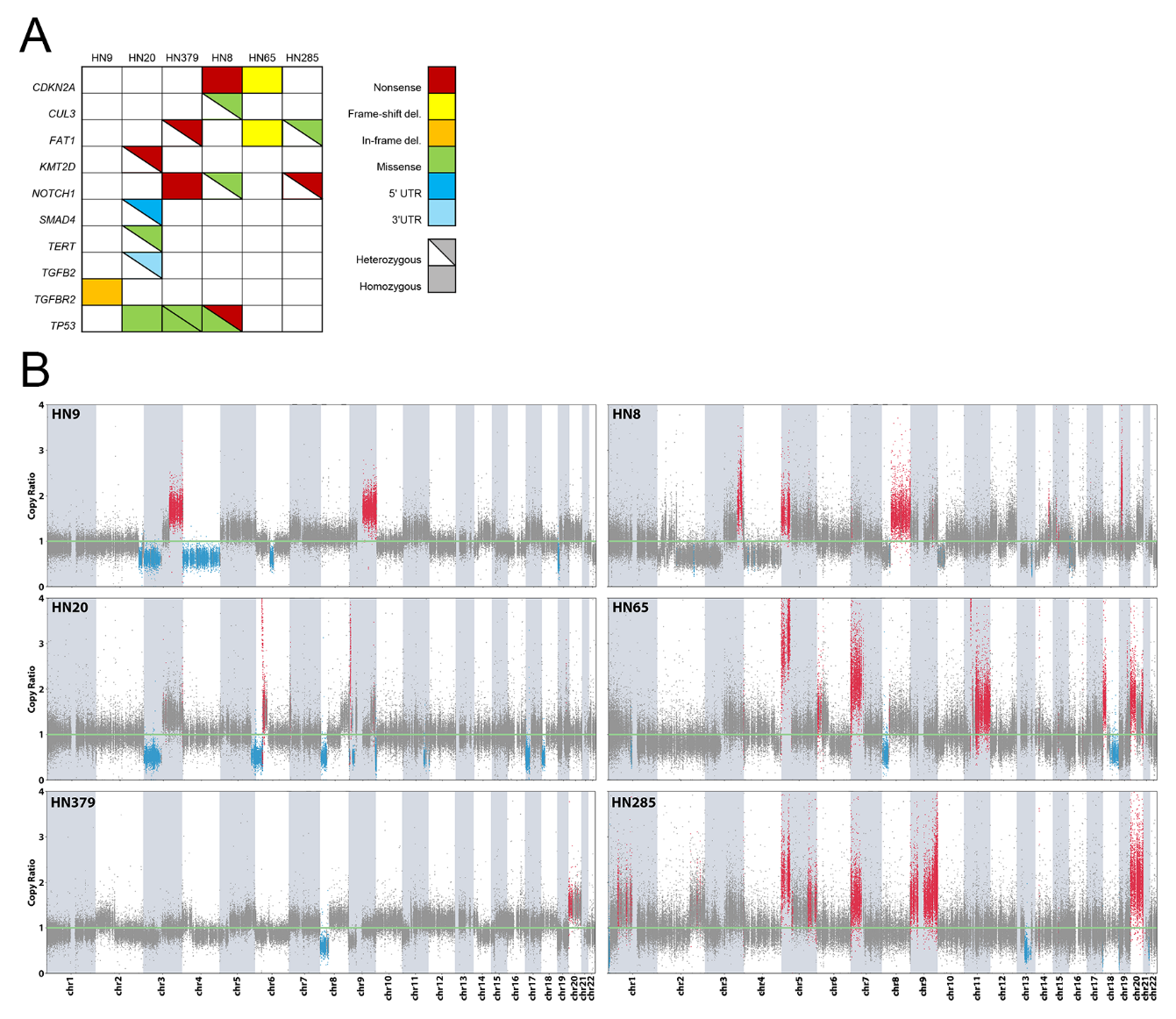
**

**Figure S4.** **HNSCC driver gene mutations and DNA copy number variations.** (A) HNSCC driver gene mutations or mutations in genes encoding TGF-beta receptors, ligands, or SMAD transcription factors identified using whole-exome sequencing. (B) Copy number ratios of HN cell lines as determined from whole-exome sequencing and compared with germline controls. Each dot represents a genomic segment (ordered by chromosome, *x*-axis), and the position of each dot on the *y*-axis represents its copy number ratio. The green line represents a copy ratio of 1, while red highlights genomic segments that exceed a ratio of 1.5 (implying copy number gain) and blue highlights genomic segments with a ratio of 0.66 and below (implying copy number loss).


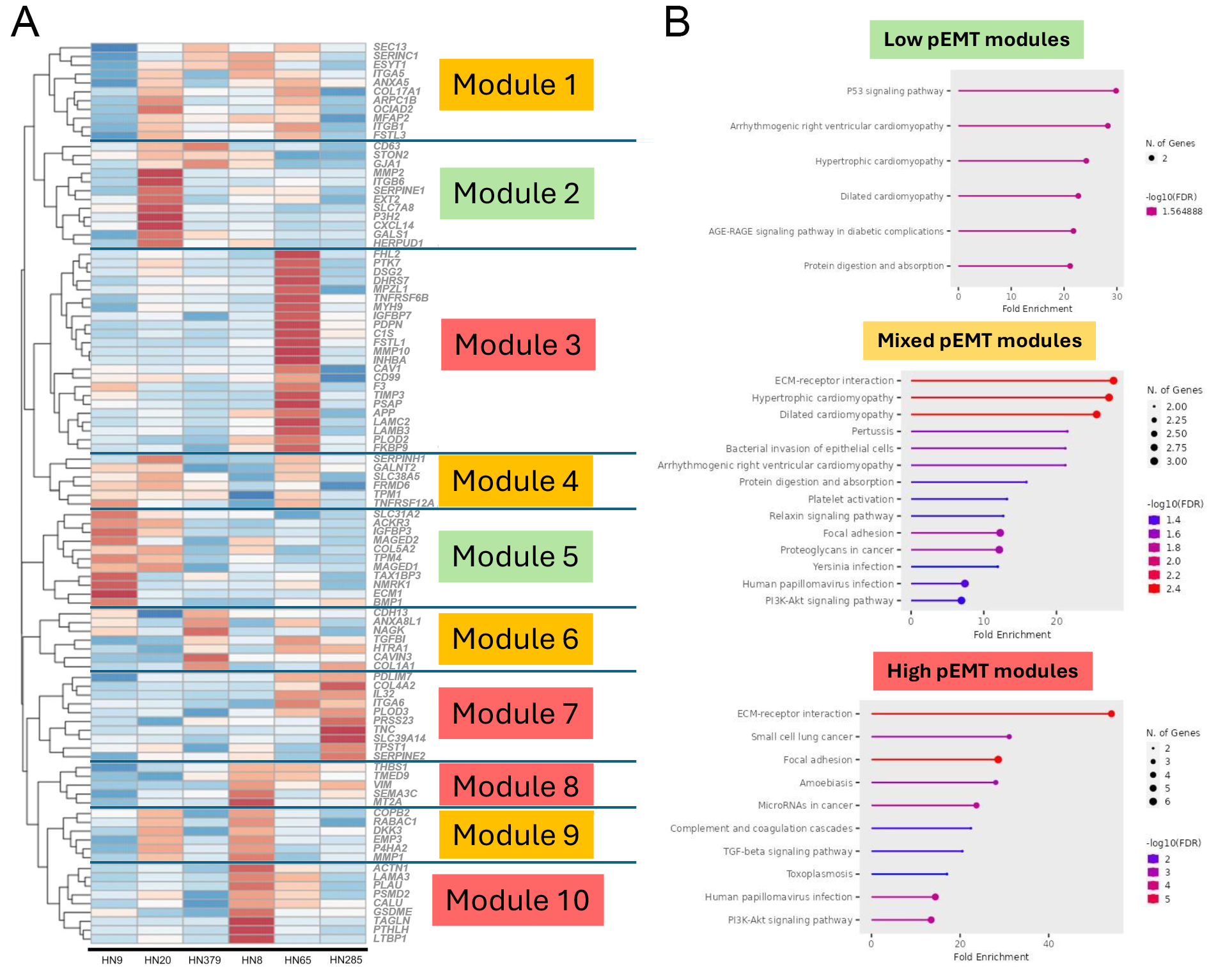


**Figure S5.** **p-EMT gene expression across HNSCC keratinocytes identifies modules of transcription.** (A) p-EMT genes can be segregated by hierarchical clustering into individual modules which can be grouped by contribution from low p-EMT HN lines (HN9, HN20, and HN379), modules 1, 4, 6, and 9; high p-EMT HN lines (HN8, HN65, and HN285), modules 3, 7, 8, and 10; or modules representing a mix of both p-EMT-low and p-EMT-high HN lines. (B) Pathway analysis of grouped modules identifies P53 signaling in low p-EMT modules, and ECM interaction, PI3K signaling, and TGF-beta signaling in mixed or high p-EMT modules.
